# Giant parathyroid adenoma presenting with severe hypercalcemia mimicking parathyroid carcinoma in a patient with chronic kidney disease: a case report

**DOI:** 10.1093/jscr/rjag548

**Published:** 2026-07-07

**Authors:** Alaa Osama Jamjoum, Hanin Alowaydhi, Mohammed Al Ameen

**Affiliations:** Endocrine Surgery Department, Ministry of Health, Second Health Cluster, Jeddah 21577, Saudi Arabia; General Surgery Department, Ministry of Health, Second Health Cluster, Jeddah 21577, Saudi Arabia; Histopathology Department, Ministry of Health, Second Health Cluster, Jeddah 21577, Saudi Arabia; Endocrine Surgery Department, Ministry of Health, Second Health Cluster, Jeddah 21577, Saudi Arabia; General Surgery Department, Ministry of Health, Second Health Cluster, Jeddah 21577, Saudi Arabia; Histopathology Department, Ministry of Health, Second Health Cluster, Jeddah 21577, Saudi Arabia; Endocrine Surgery Department, Ministry of Health, Second Health Cluster, Jeddah 21577, Saudi Arabia; General Surgery Department, Ministry of Health, Second Health Cluster, Jeddah 21577, Saudi Arabia; Histopathology Department, Ministry of Health, Second Health Cluster, Jeddah 21577, Saudi Arabia

**Keywords:** giant parathyroid adenoma, primary hyperparathyroidism, hypercalcemia, parathyroid carcinoma, intraoperative parathyroid hormone

## Abstract

Giant parathyroid adenomas (GPAs) are rare lesions that may clinically and radiologically mimic parathyroid carcinoma, particularly when associated with severe hypercalcemia. A 63-year-old male with hypertension and chronic kidney disease presented with symptomatic hypercalcemia and severe hyperparathyroidism. Laboratory evaluation revealed markedly elevated serum calcium and parathyroid hormone levels. Imaging demonstrated a large left-sided parathyroid mass suspicious for adenoma versus carcinoma. Following multidisciplinary evaluation, the patient underwent en bloc resection consisting of left thyroid lobectomy, parathyroidectomy, and central lymph node dissection. Intraoperative parathyroid hormone monitoring demonstrated a decline of more than 50%, confirming adequate excision according to the Miami criterion. Histopathological examination showed a GPA with cystic changes and no evidence of malignancy. This case highlights the diagnostic challenge of differentiating GPA from parathyroid carcinoma and emphasizes the importance of histopathological confirmation and appropriate surgical planning.

## Introduction

Primary hyperparathyroidism (PHPT) is a common endocrine disorder caused by parathyroid adenomas in ~80%–85% of cases [1, 2]. The increasing use of routine biochemical screening has shifted the presentation toward asymptomatic or mildly symptomatic disease [2].

Parathyroid adenomas are usually small lesions measuring <2 cm and weighing under 1 g. In rare cases, they enlarge significantly and are classified as giant parathyroid adenomas (GPAs), commonly defined as lesions weighing more than 3.5 g [1, 3]. GPAs are associated with markedly elevated serum calcium and parathyroid hormone (PTH) levels due to increased functional tissue mass [1, 2].

Patients may present with manifestations of hypercalcemia including fatigue, gastrointestinal symptoms, nephrolithiasis, neuropsychiatric symptoms, or a palpable cervical mass [1, 2]. A major diagnostic challenge is differentiating GPA from parathyroid carcinoma because both may present with severe hypercalcemia and large neck masses. Imaging modalities are useful for localization but cannot reliably distinguish benign from malignant lesions [1, 3]. Therefore, definitive diagnosis depends on histopathological examination.

We report a case of GPA in a patient with chronic kidney disease initially suspected to represent parathyroid carcinoma because of its size and biochemical severity.

## Case presentation

A 63-year-old male with hypertension and chronic kidney disease presented with symptomatic hypercalcemia and severe hyperparathyroidism. He complained of tremors, confusion, vomiting, and fatigue. There was no history of dysphagia, dyspnea, voice change, renal stones, fractures, or depression.

Laboratory investigations demonstrated serum calcium >4 mmol/l, PTH 221 pmol/l, and creatinine 265.33 μmol/l. Neck ultrasonography and computed tomography (CT) revealed a large left-sided parathyroid mass suspicious for adenoma versus carcinoma (Figs 1 and 2).

**Figure 1:**
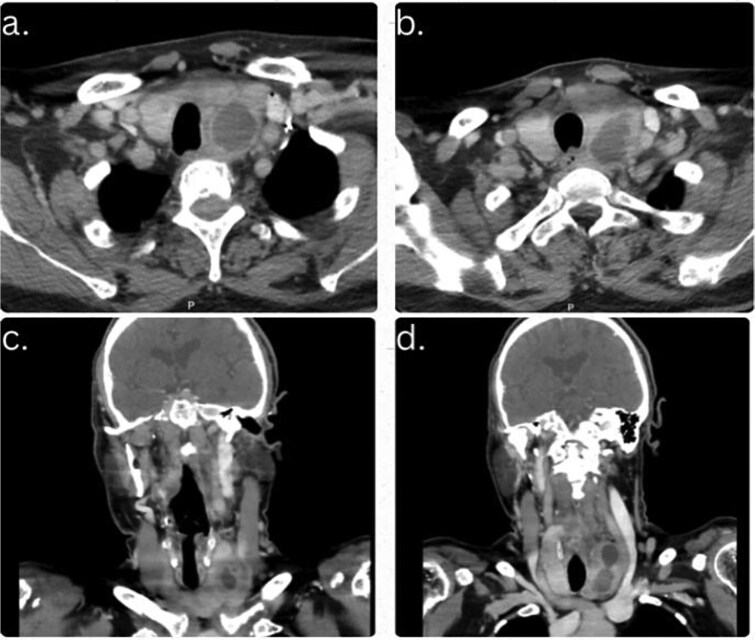
Contrast-enhanced CT neck images demonstrating a left inferior parathyroid cystic lesion measuring 4.5 × 2 × 5.2 cm (AP × TR × CC). (a and b) Axial views showing a well-defined hypodense cystic lesion posterior to the lower pole of the left thyroid lobe. (c and d) Coronal views demonstrating the extent of the retrothyroid cystic lesion without solid enhancement, calcification, or local invasion.

**Figure 2:**
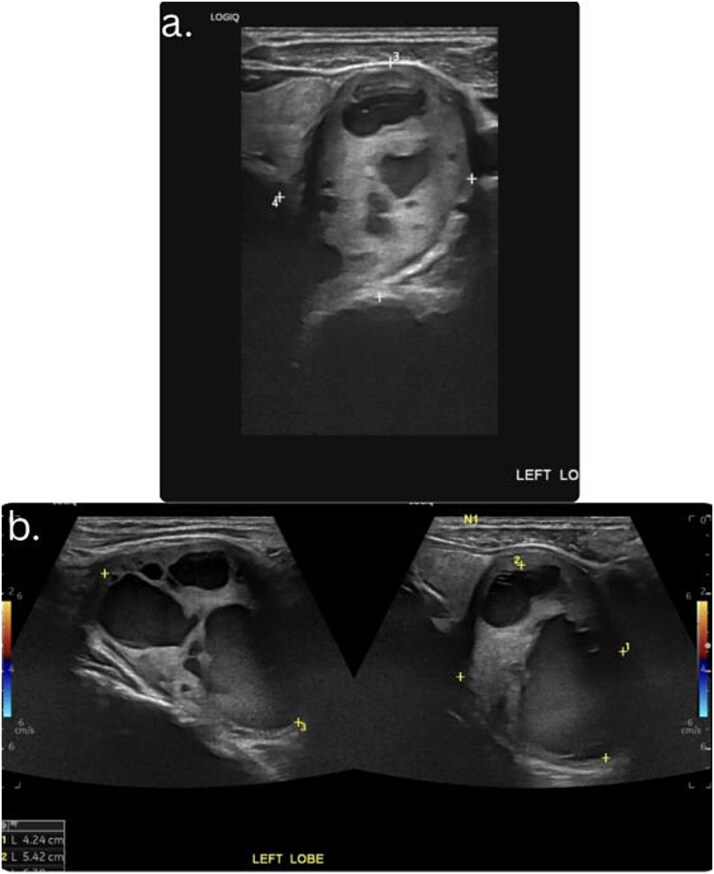
Ultrasound images of the thyroid and parathyroid region. (a and b) Longitudinal and transverse ultrasound views showing a large cystic lesion adjacent to the left thyroid region.

Following multidisciplinary discussion involving endocrinology, endocrine surgery, nephrology, and radiology teams, surgical intervention was planned because malignancy could not be excluded preoperatively. The patient underwent left thyroid lobectomy, left parathyroidectomy, and central lymph node dissection as an en bloc resection.

Intraoperatively, a large parathyroid mass adherent to the left thyroid lobe was identified without gross local invasion (Fig. 3). Intraoperative PTH monitoring demonstrated a decline >50% from the highest pre-excision value, fulfilling the Miami criterion and confirming adequate excision.

**Figure 3:**
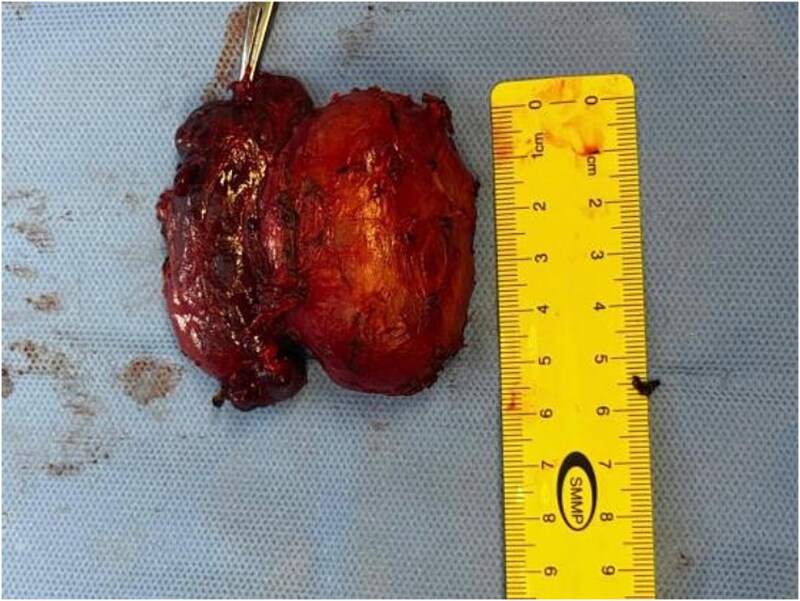
Large parathyroid mass was identified adherent to the left thyroid lobe.

The postoperative course was uneventful. The patient was monitored for hungry bone syndrome, and follow-up technetium-99 m sestamibi/SPECT imaging demonstrated no residual or ectopic parathyroid lesion.

Histopathological examination demonstrated a well-circumscribed parathyroid neoplasm composed predominantly of uniform chief cells arranged in solid sheets and nests with marked cellularity and absence of adipose tissue, consistent with parathyroid adenoma (Fig. 4). The lesion showed an intact fibrous capsule without capsular or vascular invasion. No atypical mitotic figures were identified. The thyroid lobe demonstrated multinodular goiter, while ten central lymph nodes were reactive and negative for malignancy. Overall findings were consistent with a benign GPA with cystic changes. Additional immunohistochemical studies were recommended to exclude atypical adenoma or carcinoma (Fig. 5).

**Figure 4:**
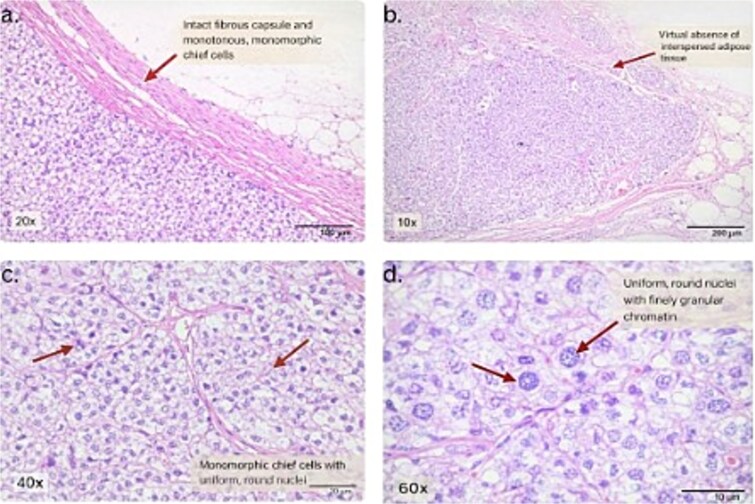
Histopathological examination of the parathyroid lesion (H&E stain). (a) Low-power view (20×) showing an intact fibrous capsule and monotonous, monomorphic chief cells. (b) Low-power view (10×) demonstrating virtual absence of interspersed adipose tissue. (c) High-power view (40×) showing monomorphic chief cells with uniform, round nuclei. (d) High-power view (60×) demonstrating uniform round nuclei with finely granular chromatin.

**Figure 5:**
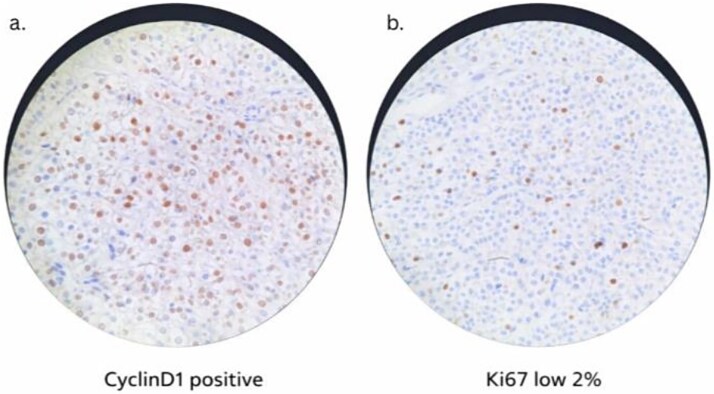
Immunohistochemical staining of the parathyroid lesion. (a) Cyclin D1 immunostain demonstrating positive nuclear expression in tumor cells. (b) Ki-67 immunostain showing a low proliferative index of ~2%. Upon the patient’s follow-up with endocrinology and surgery, his tremors and fatigue significantly improved, and no hypocalcemia symptoms were perceived. After 4 months from surgery, the patient had a normal calcium level with slightly elevated parathyroid hormone (14 pmol/l) pertaining to his chronic kidney disease, and a slightly insufficient level of vitamin D (71.68 nmol/l).

## Discussion

GPAs are rare and account for ~1.5% of parathyroid adenomas [1, 3, 4]. Compared with typical adenomas, they are associated with more severe biochemical abnormalities because of increased glandular mass [1, 5].

Differentiating GPA from parathyroid carcinoma remains challenging. Large tumor size, markedly elevated calcium levels, and severe PTH elevation may suggest malignancy but are not specific findings [5, 6]. In our patient, the lesion measured 6 cm and weighed 40.7 g, raising strong preoperative suspicion for carcinoma.

Although ultrasonography, CT, and nuclear imaging are useful for localization, they cannot reliably differentiate benign from malignant lesions [3, 5].

Histopathological examination therefore remains the gold standard for diagnosis. In this case, the absence of capsular invasion, vascular invasion, and atypical mitoses confirmed the diagnosis of benign GPA despite the lesion’s large size.

Several studies have shown that tumor size alone is not predictive of malignancy, as very large adenomas may remain histologically benign [5, 6]. The severity of biochemical abnormalities in GPA is thought to correlate with gland weight and functional cellular mass [2].

Surgical excision is the definitive treatment for GPA. Intraoperative adjuncts such as frozen section analysis and intraoperative PTH monitoring help confirm successful resection [7, 8]. According to the Miami criterion, a decline in intraoperative PTH >50% after excision indicates adequate removal of hyperfunctioning tissue [9, 10]. Our patient demonstrated a significant intraoperative PTH decline consistent with successful resection.

This case also highlights the importance of a multidisciplinary approach, particularly in patients with chronic kidney disease where severe hypercalcemia may worsen renal dysfunction. Early surgical intervention resulted in clinical and biochemical improvement.

## Conclusion

GPAs are rare causes of PHPT and may closely mimic parathyroid carcinoma clinically and biochemically. Definitive diagnosis relies on histopathological evaluation, as tumor size alone does not indicate malignancy. Surgical excision remains both diagnostic and curative, with excellent outcomes when performed promptly.

## Data Availability

All data generated or analyzed during this study are included in this published article. Additional details are available from the corresponding author upon reasonable request.
